# Development and validation of the predictive score for pediatric COVID-19 pneumonia: A nationwide, multicenter study

**DOI:** 10.1371/journal.pone.0273842

**Published:** 2022-08-29

**Authors:** Araya Satdhabudha, Chanapai Chaiyakulsil, Rattapon Uppala, Watit Niyomkarn, Prakarn Tovichien, Vasinee Norasettekul, Kanokpan Ruangnapa, Chutima Smathakanee, Bararee Choursamran, Aunya Kulbun, Rasintra Jaroenying, Harutai Kamalaporn, Tidarat Sriboonyong, Koonkoaw Roekworachai, Kanokkarn Sunkonkit, Auchara Tangsathapornpong, Pornumpa Bunjoungmanee, Wanida Pao-in, Patcharapa Thaweekul, Pichaya Tantiyavarong, Thanyarat Ratanavongkosol, Chutima Thongnual, Paskorn Sritipsukho, Jitladda Deerojanawong

**Affiliations:** 1 Department of Pediatrics, Faculty of Medicine, Thammasat University Hospital, Thammasat University, Pathumthani, Thailand; 2 Department of Pediatrics, Faculty of Medicine, Khon Kaen University, Khon Kaen, Thailand; 3 Department of Pediatrics, Faculty of Medicine, Chulalongkorn University, Bangkok, Thailand; 4 Department of Pediatrics, Faculty of Medicine Siriraj Hospital, Mahidol University, Bangkok, Thailand; 5 Department of Pediatrics, Charoenkrung Pracharak Hospital, Bangkok, Thailand; 6 Department of Pediatrics, Faculty of Medicine, Prince of Songkla University, Songkhla, Thailand; 7 Department of Pediatrics, Hat Yai Hospital, Songkhla, Thailand; 8 Department of Pediatrics, Sawanpracharak Hospital, Nakhon Sawan, Thailand; 9 Department of Pediatrics, Her Royal Highness Maha Chakri Sirindhorn Medical Center, Faculty of Medicine, Srinakharinwirot University, Nakhon Nayok, Thailand; 10 Department of Pediatrics, Faculty of Medicine, Phramongkutklao Hospital, Bangkok, Thailand; 11 Phramongkutklao Hospital Sleep Disorder Center, Faculty of Medicine, Phramongkutklao Hospital, Bangkok, Thailand; 12 Department of Pediatrics, Faculty of Medicine, Ramathibodi Hospital, Mahidol University, Bangkok, Thailand; 13 Department of Pediatrics, Nakornping Hospital, Chiangmai, Thailand; 14 Department of Pediatrics, Faculty of Medicine, Chiang Mai University, Chiang Mai, Thailand; 15 Department of Clinical Epidemiology, Faculty of Medicine, Thammasat University, Pathumthani, Thailand; 16 Center of Excellence in Applied Epidemiology, Thammasat University, Pathumthani, Thailand; Oswaldo Cruz Foundation, BRAZIL

## Abstract

**Background:**

Due to the possibility of asymptomatic pneumonia in children with COVID-19 leading to overexposure to radiation and problems in limited-resource settings, we conducted a nationwide, multi-center study to determine the risk factors of pneumonia in children with COVID-19 in order to create a pediatric pneumonia predictive score, with score validation.

**Methods:**

This was a retrospective cohort study done by chart review of all children aged 0–15 years admitted to 13 medical centers across Thailand during the study period. Univariate and multivariate analyses as well as backward and forward stepwise logistic regression were used to generate a final prediction model of the pneumonia score. Data during the pre-Delta era was used to create a prediction model whilst data from the Delta one was used as a validation cohort.

**Results:**

The score development cohort consisted of 1,076 patients in the pre-Delta era, and the validation cohort included 2,856 patients in the Delta one. Four predictors remained after backward and forward stepwise logistic regression: age < 5 years, number of comorbidities, fever, and dyspnea symptoms. The predictive ability of the novel pneumonia score was acceptable with the area under the receiver operating characteristics curve of 0.677 and a well-calibrated goodness-of-fit test (*p =* 0.098). The positive likelihood ratio for pneumonia was 0.544 (95% confidence interval (CI): 0.491–0.602) in the low-risk category, 1.563 (95% CI: 1.454–1.679) in the moderate, and 4.339 (95% CI: 2.527–7.449) in the high-risk.

**Conclusion:**

This study created an acceptable clinical prediction model which can aid clinicians in performing an appropriate triage for children with COVID-19.

## Introduction

Coronavirus disease 2019 (COVID-19) is a pandemic caused by Severe Acute Respiratory Syndrome Coronavirus 2 (SARS-COV2) that emerged in 2019. It rapidly spread from Wuhan, China, to all corners of the planet. More than 494 million cases of COVID-19 and 6 million deaths have been reported. In the pediatric context, COVID-19 cases were initially estimated to range from 0.1–15% of total cases. With new variants identified in the second half of 2021, however, the pediatric COVID-19 infection rate grew to 28% of total cases including the adult population. Severe pediatric cases and deaths have been approximately 0.6–5% and 0.3%, respectively [1–12]. Although infection rate and symptom severity are disproportionately lower in children versus adults, children are still at risk for severe disease and death due to the limitations of vaccination in young children. Children with comorbidities are also at higher risk for the development of a more severe clinical course of infection.

All confirmed COVID-19 cases in Thailand are ostensibly admitted to the healthcare system. Initially, most patients were admitted to the hospital in the pre-Delta era. Due to the increase in patients during the Delta wave, the government added external health care facilities such as field hospitals and home isolation as options for patients with mild disease. A triage system also began for patients: green (asymptomatic or mild symptoms), yellow (mild dyspnea/high-risk group with mild symptoms), red (severe pneumonia with an oxygen saturation < 96% or patients with severe symptoms requiring ventilatory or inotropic support). Green patients were admitted to the field hospitals or home isolation system, whilst yellow and red patients were hospitalized. Bedside physical examination in these patients was limited due to the enhanced personal protective equipment (PPE) and the isolation process which impeded bedside evaluation and chest auscultation. As protocol, all hospitalized patients underwent chest X-rays (CXR), but patients in home isolation underwent CXR only if exhibiting signs of lower respiratory tract infection such as dyspnea or shortness of breath.

A meta-analysis in children by Ding et al. reported that approximately 20% of asymptomatic patients had abnormal chest X-rays (CXR) [8] and another study by Liu et al. revealed that abnormal computed tomography (CT) of the chest compatible with COVID-19 could be found in asymptomatic children [13]. In this regard, this protocol might lead to excessive CXR to rule out lower respiratory tract infections in hospitalized patients but a possible under-detection of pneumonia in home isolation patients. A new predictive score derived from basic patient demographic data and clinical clues could reduce the risk of unnecessary exposure to radiation and also reduce the risk of pneumonia misdiagnosis.

Some data on risk factors for the development of severe diseases in pediatric COVID-19 has come to light. While almost all reports have been from China, Europe, and the United States, it has demonstrated that risk factors for severe diseases include underlying comorbidities, hypoalbuminemia, lymphopenia, and high acute phase reactant [1–3, 10–27]. However, much of this was determined by laboratory investigations that may not be practical for real-life clinical practice in limited-resource areas. Data has also been lacking on pediatric COVID-19 from Southeast and South Asia [4, 28]. In Thailand, there is only a single-center retrospective study looking at the epidemiology and risk factors in pneumonia development and severe pediatric COVID-19 [28]; nonetheless, the information on the pertinent risk factors was still insufficient to create any predictive score. Thus, the main objective of our study was to conduct a nationwide, multi-center study to determine the risk factors associated with the development of pneumonia in children with COVID-19, create a predictive score system, and validate this score.

## Materials and methods

### Study design

We conducted a retrospective cohort study of all pediatric patients aged from 0–15 years with PCR-confirmed COVID-19 infections between January 13^th^, 2020, and October 31^st^, 2021, who were admitted to the healthcare systems which include the hospital, field hospital, and the home isolation system at 13 centers in Thailand: 8 sites in central Thailand, 2 sites in the north, 2 sites in the south, and 1 site in the northeast. Children diagnosed with perinatal COVID-19 infection and those without CXR were excluded from the analysis. Approval for the study was granted by the Central Research Ethics Committee (CREC) of Thailand: CREC0016/2565. An online standardized database was set up using REDCap (Research Electronic Data Capture) with the main coordinating center at Thammasat University. Demographic data and clinical information were collected through a manual chart review and entered through REDCap by experienced pediatricians. Due to the study’s retrospective nature, the informed consent was waived by the CREC.

### Model development of pneumonia score

A clinical prediction model for predicting pneumonia in children with COVID-19 was created using patient data in the pre-Delta period and then using this model to externally validate the Delta population. Confirmed COVID-19 cases were classified into two groups based on the Ministry of Public Health of Thailand (MoPH) nationwide surveillance data of SARS-CoV2 variants: a pre-Delta group (diagnosed before July 1^st^, 2021) and a Delta dominant group (from July 1^st^, 2021, to October 31^st^, 2021).

Univariate analyses were used to determine potential risk factors for radiographic pneumonia at a statistical significance level of 5%. Whenever univariate statistical analyses showed the same significance level in two or more parameters that correlated with each other, the variable that provided better discriminative ability was selected. Afterward, backward and forward stepwise logistic regression was performed to acquire a final prediction model. Multivariate analyses were then performed after adjusting for statistically significant confounding factors from the univariate analysis. Clinical relevance was determined by calculating the odds ratios (ORs) with 95% confidence intervals (95% CI) and coefficient. The regression coefficients of each clinical predictor were divided by the smallest coefficient from the multivariate model and rounded to the nearest integer to transform into an item risk score. Scores from each clinical predictor were added up to obtain a total risk score. We called the new scoring system the “pneumonia score”.

### Data collection

Clinical information from both the score development cohort and validation one was collected: demographic data, underlying medical conditions, nutritional status, clinical history, initial vital signs, clinical symptoms, CXR findings, disease severity, and clinical outcome.

### Operational definition

For children < 5 years of age, obesity was defined as weight-for-height > 3 standard deviations (SD) above the World Health Organization (WHO) Child Growth Standard median [29, 30]. Children and adolescents aged between 5–15 years were defined as obese if BMI-for-age was > 2 SD above the WHO Growth Reference Median [29, 31]. Tachycardia and tachypnea for age were defined using WHO criteria [32]. Desaturation was defined as oxygen saturation < 95%. Pneumonia was defined as patients with clinical signs and symptoms of lower respiratory tract infection such as dyspnea, tachypnea, and desaturation with abnormal CXR or asymptomatic patients with abnormal CXR. All CXR were reviewed by radiologists and/or pediatric pulmonologists to ensure accuracy. Disease severity categorization was based on the National Institutes of Health (NIH) [33]. Five categories were defined: asymptomatic, mild (mild symptoms without pneumonia), moderate (pneumonia without desaturation or no symptoms but abnormal CXR), severe (pneumonia with SpO_2_ < 94%), or critically ill (patients requiring mechanical ventilation or vasoactive medication; acute respiratory distress syndrome; or septic shock). Immunocompromised children were defined as children who underwent bone marrow or solid organ transplantation, children who are currently taking immunosuppressives medications, children who are undergoing cancer treatment, children with primary immunodeficiencies, and children with advanced or untreated HIV infection [34].

### Statistical analyses

Data were analyzed using STATA for Windows v14.0 (StataCorp LLC, Texas, USA). Clinical characteristics in terms of continuous data were reported as mean and standard deviation or median with interquartile range (IQR) and categorical variables were reported as the frequency with percentage. One-way ANOVA, Wilcoxon rank-sum test, and Kruskal-Wallis test were used to compare continuous data. Categorical data analysis was conducted using a Chi-square test adjusted for multiple comparisons.

The external validity of the developed model was evaluated in terms of both discrimination and calibration. The discriminative ability of the model was determined using Mann–Whitney U test. Predictive accuracy was presented as the area under the receiver operating characteristic (AuROC) curve, along with 95% CI. The calibration plot was illustrated, and the Hosmer–Lemeshow test was conducted to demonstrate the results of model calibration. Pneumonia scores were categorized into three risk levels: high, moderate, and low risk. The predictive ability of each risk score level was calculated and presented as a positive likelihood ratio and 95% CI.

## Results

### Overall patient characteristics

A total of 4,628 confirmed pediatric COVID-19 cases were enrolled during the study period. Approximately 3,932 patients (85%) had CXR and were included in the development and validation analysis. The score development cohort consisted of 1,076 patients in the pre-Delta era, and the validation cohort included 2,856 patients from the Delta era. Weight-for-height was obtained in 2844 cases due to the difficulty in obtaining height with the isolation process at some centers. The proportion of children with obesity did not differ among groups (18.5% VS 20.7%; *p* = 0.174).

The demographic data of children within all cohorts were described in Table 1. There were significantly fewer patients who were asymptomatic in the validation cohort (14.6%) when compared to the score development cohort (26.6%) (*p <* 0.001). There was a significantly higher proportion of pneumonia in the validation cohort (32.7% VS 28.8%; *p =* 0.020). Nevertheless, there were no significant differences in the utilization of respiratory support, intensive care admission, and mortality rates among the cohorts.

**Table 1:** Demographic data and clinical characteristics in different periods of 3,932 confirmed pediatric COVID-19 cases.

Variables	All cases N = 3,932	Category by variant		*p*-value
		Pre-Delta N = 1,076	Delta N = 2,856	
**Male gender (N; %)**	2,015 (51.3%)	540 (50.2%)	1,475 (51.7%)	0.414
**Age group (N; %)**				
• < 1 year	197 (5.0%)	36 (3.3%)	161 (5.7%)	0.003
• 1 year—< 5 years	1,400 (35.6%)	385 (35.8%)	1,015 (35.5%)	0.888
• 5 years—< 10 years	1,133 (28.8%)	313(29.1%)	820 (28.7%)	0.816
• 10 years—15 years	1,202 (30.6%)	342 (31.8%)	860 (30.1%)	0.310
**Region (N; %)**				
• Central	2,775 (70.6%)	850 (79.0%)	1,925 (67.4%)	< 0.001
• Northeast	349 (8.9%)	21 (1.9%)	328 (11.5%)	< 0.001
• South	643 (16.3%)	174 (16.2%)	469 (16.4%)	0.850
• North	165 (4.2%)	31 (2.9%)	134 (4.7%)	0.012
**Obese (N; %)**	N = 2,844	N = 830	N = 2014	
	572 (20.1%)	154 (18.5%)	418 (20.7%)	0.174
**Comorbidities (N; %)** *				
• None	3,561 (90.6%)	977 (90.9%)	2,584 (90.5%)	0.697
• Chronic lung disease	17 (0.4%)	4 (0.4%)	13 (0.5%)	0.722
• Allergic disease	180 (4.6%)	43 (4.0%)	137 (4.8%)	0.284
• Cardiovascular disease	31(0.8%)	8 (0.7%)	23 (0.8%)	0.845
• Neurologic disease	32 (0.8%)	10 (0.9%)	22 (0.8%)	0.621
• Endocrine disease	17 (0.4%)	7 (0.7%)	10 (0.4%)	0.201
• Hypertension	9 (0.2%)	3 (0.3%)	6 (0.2%)	0.688
• GI disease	9 (0.2%)	4 (0.4%)	5 (0.2%)	0.250
• Chronic renal disease	8 (0.2%)	3 (0.3%)	5 (0.2%)	0.520
• Hematologic disease	55 (1.4%)	12 (1.1%)	43 (1.5%)	0.353
• Oncological	7 (0.2%)	2 (0.2%)	5 (0.2%)	0.943
• Genetic and DBP	42 (1.1%)	12 (1.1%)	30 (1.1%)	0.860
**Number of comorbidities (N;%)**				0.929
• No	3,563 (90.6%)	978 (90.9%)	2,585 (90.5%)	
• 1	334 (8.5%)	89 (8.3%)	245 (8.6%)	
• ≥ 2	35 (0.9%)	9 (0.8%)	26 (0.9%)	
**Symptoms (N; %)**				
• Asymptomatic	960 (24.4%)	394 (36.6%)	566 (19.8%)	< 0.001
• Fever	1,871 (47.6%)	314 (29.2%)	1,557 (54.5%)	< 0.001
• Dyspnea	105 (2.7%)	20 (1.9%)	85 (3.0%)	0.053
• Chest pain	22 (0.6%)	6 (0.6%)	16 (0.6%)	0.992
**Clinical signs (N; %)**				
• High fever (BT ≥ 38.5°C)	782 (19.9%)	123 (11.4%)	659 (23.1%)	< 0.001
• Tachypnea for age	155 (3.9%)	62 (5.8%)	93 (3.3%)	< 0.001
• Desaturation	30 (0.8%)	11 (1.0%)	19 (0.7%)	0.250
**Abnormal CXR (N; %)**	1243 (31.6%)	310 (28.8%)	933 (32.7%)	0.020
**Peak severity (N; %)**				< 0.001
• Asymptomatic	702 (17.8%)	286 (26.6%)	416 (14.5%)	
• Mild	1,976 (50.3%)	479 (44.5%)	1,497 (52.4%)	
• Moderate	1,202 (30.6%)	306 (28.4%)	896 (31.4%)	
• Severe	40 (1.0%)	4 (0.4%)	36 (1.3%)	
• Critically ill	12 (0.3%)	1 (0.1%)	11 (0.4%)	
**Respiratory support (N; %)**	68 (1.7%)	13 (1.2%)	55 (1.9%)	0.474
**ICU admission (N; %)**	26 (0.7%)	5 (0.5%)	21 (0.8%)	0.249
**Death (N; %)**	4 (0.1%)	1 (0.1%)	3 (0.1%)	0.915

*Some patients can have more than one underlying condition. Allergic disease = asthma, allergic rhinitis, and other allergic diseases; Developmental disease = autism, attention-deficit-hyperactivity disorder

CXR = Chest X-ray; DBP = developmental and behavioral pediatrics

### Model generation

In the development cohort, pneumonia was diagnosed in 310 patients (28.8%); 87 children (28.1%) were asymptomatic. There was a significantly higher proportion of children < 5 years old who developed pneumonia versus older ones (52.9% VS 33.5%; *p <* 0.001). Children with comorbidities (12.2% VS 7.8%; *p =* 0.011) and children exhibiting dyspnea symptoms (4.2% VS 0.9%; *p <* 0.001) were more likely to have pneumonia. There were a significantly higher proportion of children with pneumonia in children who exhibited the symptoms of fever (38.7% VS 25.3%; *p <* 0.001) and cough (46.8% VS 38.8%; *p =* 0.016). Children with pneumonia were more likely to be tachycardic for age and have desaturation when compared to children without pneumonia. Gender, obesity status, and immunocompromised status were not associated with pneumonia. Associated risk factors for the development of radiographic pneumonia as determined by univariate analyses are given in Table 2.

**Table 2:** Univariate analysis of risk factors in the development of radiographic pneumonia for 1,076 confirmed pediatric COVID-19 cases in the pre-Delta period.

Variables	Pneumonia N = 310	No pneumonia N = 766	OR (95%CI)	*p*-value
**Male gender (N; %)**	153 (49.3%)	387 (50.5%)	0.954 (0.733–1.243)	0.729
**Age group (N; %)**				< 0.001*
• < 1 year	12 (3.9%)	24 (3.1%)	1.984 (0.937–4.203)	0.068
• 1 year—< 5 years	152 (49.0%)	233 (30.4%)	2.589 (1.801–3.679)	< 0.001
• 5 years—< 10 years	63 (20.3%)	250 (32.7%)	Reference	-
• 10 years—< 15 years	83 (26.8%)	259 (33.8%)	1.271 (0.877–1.844)	0.204
**Obese (N; %)**	44 (14.2%)	110 (14.4%)	0.945 (0.642–1.392)	0.803
**Numbers of comorbidities (N; %)**				0.011*
• No	272 (87.8%)	706 (92.2%)	Reference	-
• 1	32 (10.3%)	57 (7.4%)	1.457 (0.923–2.298)	0.103
• ≥ 2	6 (1.9%)	3 (0.4%)	5.191 (1.282–21.017)	0.009
**Immunocompromised host (N; %)**	6 (1.9%)	5 (0.6%)	2.040 (0.564–7.376)	0.267
**Symptoms (N; %)**				
• Asymptomatic	87 (28.1%)	307 (40.1%)	0.583 (0.437–0.779)	< 0.001
• Fever	120 (38.7%)	194 (25.3%)	1.862 (1.403–2.472)	< 0.001
• Cough	145 (46.8%)	297 (38.8%)	1.388 (1.062–1.812)	0.016
• Rhinorrhea	77 (24.8%)	180 (23.5%)	1.076 (0.791–1.462)	0.641
• Nasal congestion	6 (1.9%)	28 (3.7%)	0.520 (0.213–1.270)	0.144
• Sore throat	25 (8.1%)	74 (9.7%)	0.820 (0.510–1.318)	0.412
• Dyspnea	13 (4.2%)	7 (0.9%)	4.746 (1.863–12.085)	< 0.001
• Chest pain	3(1.0%)	3 (0.4%)	2.485 (0.498–12.403)	0.251
**Clinical signs (N; %)**				
• High fever (BT ≥ 38.5°C)	26 (8.4%)	16 (2.1%)	4.291 (2.252–8.178)	< 0.001
• Tachycardia for age	12 (3.9%)	19 (2.5%)	1.583 (0.758–3.304)	0.217
• Tachypnea for age	28 (9.0%)	34 (4.4%)	2.138 (1.269–3.599)	0.003
• Desaturation	6 (1.9%)	5 (0.7%)	3.013 (0.910–9.974)	0.057

*By likelihood ratio test

CXR = Chest X-ray; 95% CI = 95% confidence interval

Multivariate analyses were then performed after adjusting for statistically significant confounding factors from the univariate analysis. Four predictors remained after backward and forward stepwise logistic regression: age < 5 years, number of comorbidities, fever, and dyspnea symptoms. Coefficients in terms of the point estimate, odds ratios, and 95% CI were created for the statistically significant clinical variables to generate a score for each parameter. The scores from each parameter were then added to create a sum for the predictive score with a maximum of 16. Details are in Table 3.

**Table 3:** Clinical predictive score for pneumonia.

Variables	Coefficient	95% CI	*p*-value	Score
**Age < 5 year**	0.861	0.585–1.137	<0.001	3
**Numbers of comorbidities**			0.025	
1	0.349	-0.136–0.836		1
>2	1.655	0.204–2.207		5
**High fever (≥ 38.5°C)**	1.415	0.752–2.078	<0.001	4
**Dyspnea**	1.222	0.237–2.2.7	0.015	4

95% CI = 95% confidence interval

### Model validation

During the validation period, a total of 933 patients (32.7%) were diagnosed with pneumonia. A total of 113 patients with pneumonia (12.1%) were asymptomatic. The discriminative ability of the pneumonia score, which ranged from 0 to 16, was presented with the ROC curve as shown in S1 Fig. The score predicted abnormal CXR in pediatric patients with an AuROC curve of 0.677 (95% CI: 0.656–0.686; *p <* 0.001). The pneumonia scores were considered a well-calibrated scoring system, given that the Hosmer–Lemeshow test demonstrated the goodness-of-fit test with the *p*-value of 0.098. The calibration plot is illustrated in S2 Fig. The pneumonia score was categorized into three risk groups: low (< 3), moderate (3 to 7), and high (> 7) to facilitate clinical interpretation. The positive likelihood ratio (LHR+) for pneumonia was 0.544 (95%CI: 0.491–0.602) in the low-risk category, 1.563 (95% CI: 1.454–1.679) in the moderate group, and 4.339 (95% CI: 2.527–7.449) in the high-risk category (Table 4).

**Table 4:** Probability categories and the predictive ability.

Probability categories	Score	Pneumonia; N = 933 (%)	Non-pneumonia N = 1923 (%)	LHR+	95% CI
Low	< 3	294 (31.5%)	1,114 (57.9%)	0.544	0.491–0.602
Medium	3–7	599 (64.2%)	790 (41.1%)	1.563	1.454–1.679
High	>7	40 (4.3%)	19 (0.9%)	4.339	2.527–7.449

95% CI = 95% confidence interval; LHR + = positive likelihood ratio

## Discussion

This study was the first in Thailand to develop and validate a predictive score for pneumonia prediction in children with COVID-19. By using this new predictive score, the aim was to reduce both overuse and underuse of CXR in pneumonia diagnosis. An appropriate triage system is ideal for both patient care and economic/budget considerations, especially where there are limited resources and medical access.

As mentioned, bedside evaluation and physical examination could be extremely limited during patient isolation. Based on the risk score, we were able to categorize children into low risk, intermediate-risk, and high risk for having pneumonia. With the low number of patients with pneumonia in the high-risk group, overuse of CXR was deemed to be as low as 0.9%. Furthermore, a high LHR+ suggested that CXR should be performed in children in high-risk groups. Our study revealed that 20.8% of asymptomatic children during the pre-Delta period and 22.1% during the Delta one had abnormal CXR. These results were comparable with the previous study by Ding et al. which demonstrated pneumonia in 20% of asymptomatic children [8]. In the low-risk group, the results demonstrated that approximately 31.5% of patients had pneumonia. Nevertheless, with the low pneumonia score, most children were either asymptomatic or presented with only mild symptoms, which did not require medical intervention. The decision to perform CXR on low-risk patients might not be cost-effective. Nevertheless, patient monitoring remains essential, and CXR should be performed when the patients exhibit more clinical characteristics which constitute a higher pneumonia score.

To date, only one large, observational cohort study in Brazil was conducted to create a clinical prediction model in children with COVID-19. Despite the difference in the endpoint, which aimed to predict death rather than pneumonia, the study by Oliveira et al. revealed similar results: children with more respiratory symptoms and comorbidities were more likely to have negative outcomes [35]; this coincides with a systematic review by Tsankov et al. [36]. Similar to an Asian multinational study by Wong et al. and Bellino et al. in Italy, our study also found younger children were more likely to have a more severe clinical course [4, 22].

Our study was the first large, nationwide, multicenter study of pediatric COVID-19 from 13 different centers across Thailand, which is a major strength. Unlike other nations, Thailand attempted to admit all patients into the healthcare system, which led to the founding of field hospitals and our home isolation system. Due to this national policy, we were able to obtain large amounts of epidemiological data not only from hospitalized children but also from children in field hospitals and home isolation. With this large and robust clinical dataset, we created a prediction score with a well-fitted calibration model and validated cohort to be used across the country, regardless of region. Different regions of Thailand have certain disparities in terms of access to medical attention. There is also a limited number of pediatricians in rural areas. Therefore, by using our model with its acceptable discrimination power and accuracy in predicting pneumonia, a triage system in these less privileged regions can be set up and provide helpful clinical assistance; this could also aid in public policy decision-making [37].

Nevertheless, several limitations must be outlined. Firstly, due to the retrospective nature of the study and limitation in obtaining the height of children in isolation, weight-for-height was obtained in 2844 cases (72% of the cohort). Despite the missing data, the remaining population was adequately powered for risk factor determination for pneumonia. In these patients, obesity was not associated with pneumonia, unlike for adults, which is in accordance with several other pediatric studies [1, 10–12, 14, 16–17].

Secondly, due to the low prevalence of severe disease and deaths in children with COVID-19 in Thailand, we were unable to create a prediction model for severe disease. Thus, we used a diagnosis of pneumonia, classified as moderate severity, as a surrogate. Another limitation worth mentioning is that this study was conducted when the pediatric vaccination rollout was still limited; thus, we were unable to include vaccination in the predictive score calculation. Another validation study might be warranted when vaccination coverage in children is higher. Finally, our study was conducted during the pre-Delta and the Delta dominant eras of COVID-19, so we were unable to make any conclusions on the effectiveness of its discrimination power on the future variants of COVID-19.

## Conclusion

This study created an acceptable clinical prediction model to predict pneumonia for children with COVID-19. Thus, by using this clinical prediction score, children can be triaged more appropriately and received improved medical care.

## Supporting information

S1 Fig Receiver-operating characteristic curve of the risk score to predict pneumonia. (TIF)

S2 Fig Calibration plot of pneumonia score. Pr (obs_pneumonia) = predicted risk; obsrisk = observed risk.
(TIF)
