# Supplementary figures and images for: Development and validation of the predictive score for pediatric COVID-19 pneumonia: A nationwide, multicenter study
Source: PLoS One. 2022 Aug 29;17(8):e0273842. doi: 10.1371/journal.pone.0273842 (PMC9423652; doi:10.1371/journal.pone.0273842)

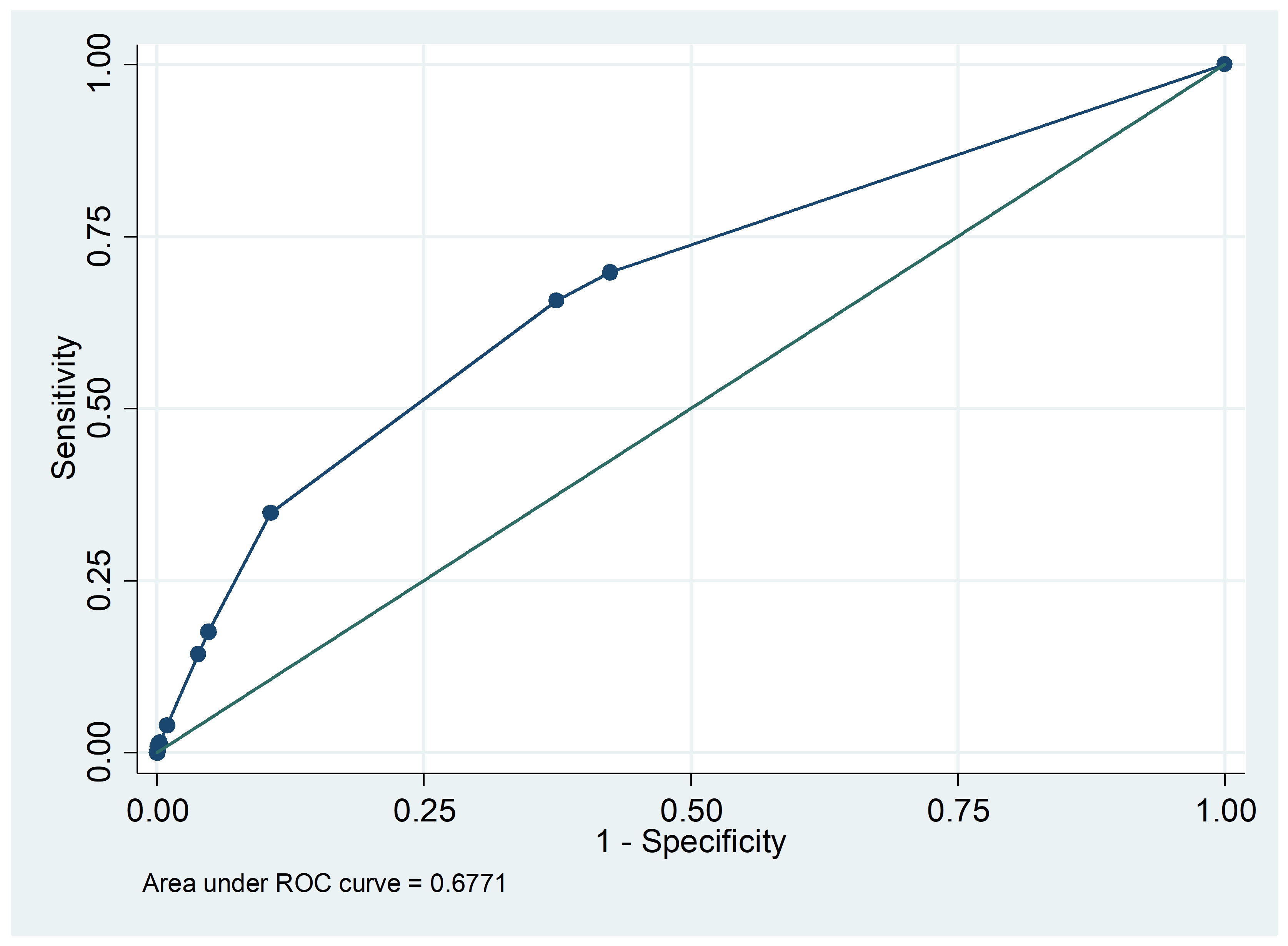

Supplement: S1 Fig — (TIF) [file pone.0273842.s001.tif]

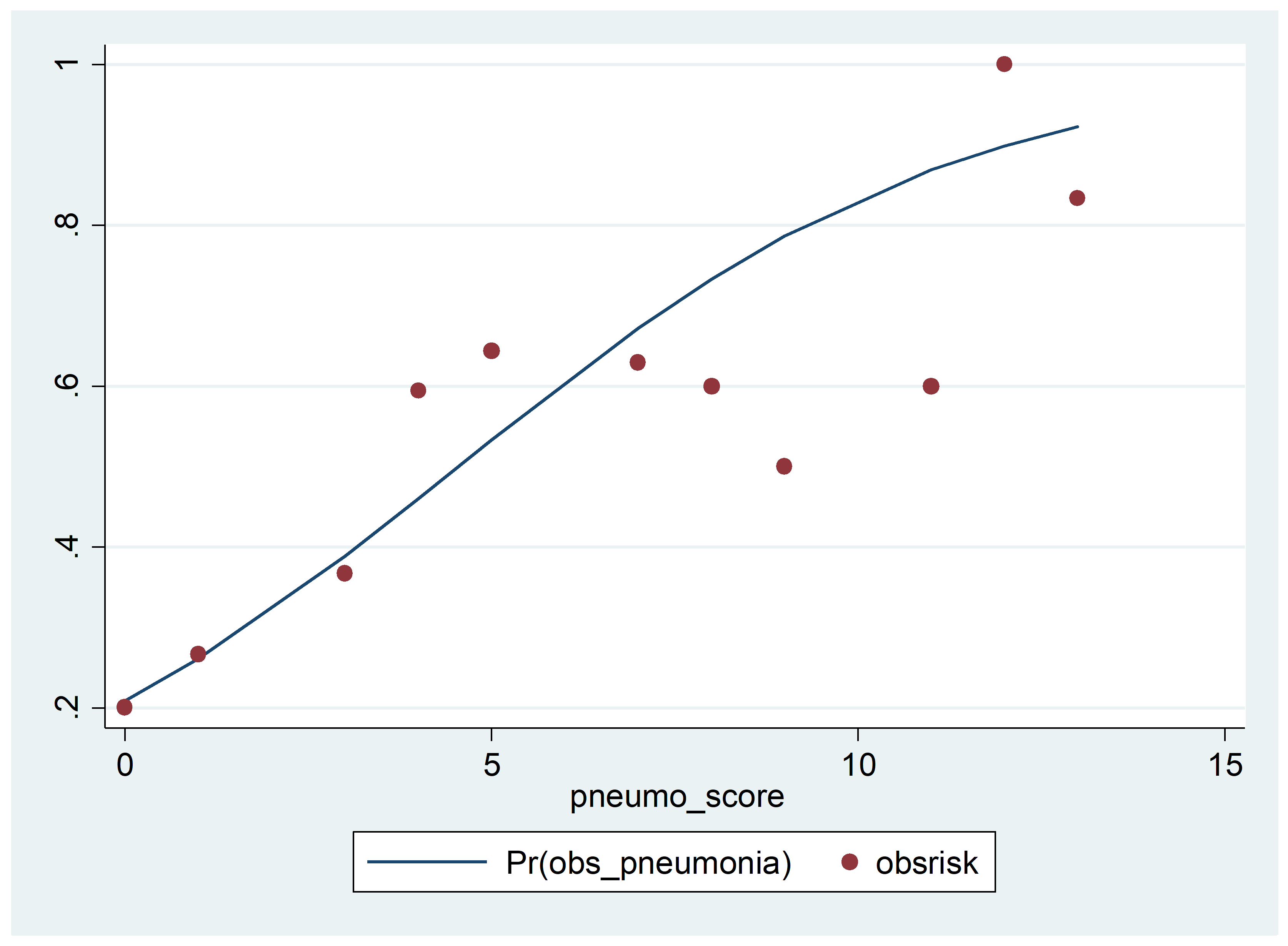

Supplement: S2 Fig — Pr (obs_pneumonia) = predicted risk; obsrisk = observed risk. (TIF) [file pone.0273842.s002.tif]
